# Venous thrombosis and predictors of relapse in eosinophil-related diseases

**DOI:** 10.1038/s41598-021-85852-9

**Published:** 2021-03-18

**Authors:** Valériane Réau, Alexandre Vallée, Benjamin Terrier, Aurélie Plessier, Noémie Abisror, Félix Ackermann, Ruben Benainous, Gérôme Bohelay, Marie-Laure Chabi-Charvillat, Divi Cornec, Anne-Claire Desbois, Stanislas Faguer, Nathalie Freymond, Antoine Gaillet, Mohamed Hamidou, Martin Killian, Sylvain Le Jeune, Anne Marchetti, Guy Meyer, Francisco Osorio-Perez, Kewin Panel, Pierre-Emmanuel Rautou, Julien Rohmer, Nicolas Simon, Colas Tcherakian, Marc Vasse, Elina Zuelgaray, Guillaume Lefevre, Jean-Emmanuel Kahn, Matthieu Groh

**Affiliations:** 1 Department of Internal and Geriatric Medicine, Henri Mondor Hospital, Assistance Publique-Hôpitaux de Paris, Créteil, France; 2 National Reference Center for Hypereosinophilic Syndromes, CEREO, France; 3 Department of Clinical Research and Innovation (DRCI), Hôpital Foch, 92150 Suresnes, France; 4 Department of Internal Medicine, National Referral Center for Systemic and Autoimmune Diseases, Cochin Hospital, Assistance Publique-Hôpitaux de Paris, Paris, France; 5 Department of Hepatology, Beaujon Hospital, Assistance Publique-Hôpitaux de Paris, Clichy, France; 6 Department of Internal Medicine, Saint Antoine Hospital, Assistance Publique-Hôpitaux de Paris, Paris, France; 7 Department of Internal Medicine, Hôpital Foch, 40, rue Worth, 92151 Suresnes Cedex, France; 8 Department of Internal Medicine, Avicenne Hospital, Assistance Publique-Hôpitaux de Paris, Bobigny, France; 9 Department of Dermatology, Avicenne Hospital, Assistance Publique-Hôpitaux de Paris, Bobigny, France; 10 Department of Radiology, Foch Hospital, Suresnes, France; 11 Department of Rheumatology, Brest University Hospital, Brest, France; 12 Department of Internal Medicine, Pitié-Salpêtrière Hospital, Assistance Publique-Hôpitaux de Paris, Paris, France; 13 Department of Nephrology, Toulouse University Hospital, Toulouse, France; 14 Department of Pulmonology, Lyon University Hospital, Lyon, France; 15 Department of Internal Medicine, Hôtel-Dieu University Hospital, Nantes, France; 16 Department of Internal Medicine, Saint-Etienne University Hospital, Saint-Etienne, France; 17 Department of Dermatology, Lyon-Sud Hospital, Pierre-Bénite, France; 18 Pulmonology and Intensive Care Service, Georges Pompidou European Hospital, Assistance Publique-Hôpitaux de Paris, Paris, France; 19 Department of Internal Medicine, Dax-Côte D’Argent Hospital, Dax, France; 20 Department of Internal Medicine, Grenoble Alpes University Hospital, Grenoble, France; 21 Department of Pulmonology, Foch Hospital, Suresnes, France; 22 Department of Clinical Biology, Foch Hospital, Suresnes, France; 23 UMR-S INSERM 1176, Université Paris-Saclay, Le Kremlin-Bicêtre, France; 24 Department of Dermatology, Saint Louis, Hospital, Assistance Publique-Hôpitaux de Paris, Paris, France; 25 Department of Internal Medicine, Lille University Hospital, Lille, France; 26 Department of Internal Medicine, Ambroise Paré Hospital, Assistance Publique-Hôpitaux de Paris, Boulogne-Billancourt, France

**Keywords:** Medical research, Risk factors

## Abstract

Eosinophils have widespread procoagulant effects. Eosinophilic cardiovascular toxicity mostly consists of endomyocardial damage or eosinophilic vasculitis, while reported cases of venous thrombosis (VT) are scarce. We aimed to report on the clinical features and treatment outcomes of patients with unexplained VT and eosinophilia, and to identify predictors of relapse. This retrospective, multicenter, observational study included patients aged over 15 years with VT, concomitant blood eosinophilia ≥ 1G/L and without any other moderate-to-strong contributing factors for VT. Fifty-four patients were included. VT was the initial manifestation of eosinophil-related disease in 29 (54%) patients and included pulmonary embolism (52%), deep venous thrombosis (37%), hepatic (11%) and portal vein (9%) thromboses. The median [IQR] absolute eosinophil count at VT onset was 3.3G/L [1.6–7.4]. Underlying eosinophil-related diseases included FIP1L1-PDGFRA-associated chronic myeloid neoplasm (n = 4), Eosinophilic Granulomatosis with Polyangiitis (n = 9), lymphocytic (n = 1) and idiopathic (n = 29) variants of hypereosinophilic syndrome. After a median [IQR] follow-up of 24 [10–62] months, 7 (13%) patients had a recurrence of VT. In multivariate analysis, persistent eosinophilia was the sole variable associated with a shorter time to VT relapse (HR 7.48; CI95% [1.94–29.47]; p = 0.015). Long-term normalization of eosinophil count could prevent the recurrence of VT in a subset of patients with unexplained VT and eosinophilia ≥ 1G/L.

## Introduction

Blood and/or tissue eosinophilia are reported in numerous conditions including allergic, infectious, inflammatory and neoplastic disorders^1,2^. Whatever the underlying disease, eosinophil-related clinical manifestations are heterogeneous and include tissue fibrosis or thrombosis within involved organs. To date, reports of eosinophilic cardiovascular toxicity mostly consist of endomyocardial damage (occasionally with intra-cardiac thrombi) occurring in patients with chronic helminthiasis, hypereosinophilic syndrome (HES) or eosinophilic granulomatosis with polyangiitis (EGPA, formerly Churg-Strauss syndrome)^3,4^. Moreover, we recently provided evidence supporting arterial eosinophil-related toxicity in patients with either single-organ or systemic eosinophilic vasculitis (in the absence of polyarteritis nodosa or EGPA)^5^, including cases of thromboangiitis obliterans-like disease^6^.

In patients with hypereosinophilia (HE), the occurrence of venous thrombosis (VT) is considered to be an HES-defining feature according to the latest classification criteria for eosinophilic disorders and related syndromes established by the International Cooperative Working Group on Eosinophil Disorders (ICOG-Eo)^1^. However, cases of VT occurring in the setting of eosinophil-related diseases have seldom been reported, with only 5 cases in the largest multidisciplinary international collaborative series of 188 HES patients (including all disease subtypes)^7^. Moreover, HE is not listed as a predisposing factor for venous thromboembolism (VTE) (according to the European Society of Cardiology) and there are currently no guidelines for the management of VT occurring in the setting of HE^8^.

In this nationwide retrospective study, we aimed to perform a comprehensive analysis of the clinical picture and treatment outcomes of patients with new-onset VT and eosinophilia (whatever the underlying disease, but in the absence of major predisposing factors for VT), and to identify predictors of relapse.

## Methods

### Study design and inclusion criteria

We conducted a retrospective, multicenter, observational study involving collaborative networks (National Reference Center for Hypereosinophilic Syndromes, CEREO; Investigation Network On Venous Thrombo-Embolism, INNOVTE). Inclusion criteria were: (i) age ≥ 15 years; (ii) at least one imaging-confirmed VT event (whatever the site, with the exclusion of retinal vein occlusion, superficial venous thrombosis and VT secondary to a locoregional septic or neoplastic process); (iii) absolute eosinophilia count (AEC) ≥ 1G/L at VT occurrence. Exclusion criteria were either prior history of VT, hereditary thrombophilia, any condition, comorbidity or concomitant treatment leading to acquired thrombophilia, or any other major (relative risk (RR) > 10) transient or reversible predisposing factor for VTE according to the European Society of Cardiology and European Respiratory Society (a comprehensive list of exclusion criteria is provided in the Supplementary Appendix)^8^.

### Baseline measurements

All cases were reviewed by the investigators (VR, MG) taking into account the entire follow-up. Using a standardized case report form, demographic (including minor or moderate risk factors for VTE as reported previously)^8^, clinical, laboratory and imaging findings at the time of VT and during follow-up were retrospectively collected. For each patient, the underlying process underpinning blood HE was assessed according to the International Cooperative Working Group on Eosinophil Disorders (ICOG-Eo) terminology^1^ and considered as either clonal (*i.e.* neoplastic, including *FIP1L1-PDGFRA* myeloid neoplasm with eosinophilia), reactive (including all conditions e.g. parasitic infections, adverse drug reactions or neoplastic diseases that lead to the production of Th2-related cytokines and thereby to non-clonal HE), overlapping (when embodied in the spectrum of autoimmune diseases, e.g. EGPA^9^, IgG4-related diseases^10^ or bullous pemphigoid^11^), or idiopathic.

### Outcomes

During follow-up, studied outcomes included the recurrence of VT (defined as new-onset symptoms confirmed by imaging examinations, whether in the same or a distinct anatomical site from the initial episode), major bleeding events and vascular relapse (consisting of either VT recurrence or new-onset arterial thrombosis) and death. “Persistent eosinophilia” and “long-term anticoagulant therapy” were defined as an AEC > 0.5G/L and as continued (> 6 months) anticoagulant therapy at the time of either vascular relapse (for relapsing patients) or at follow-up (for non-relapsing patients), respectively.

### Statistical analyses

Patient characteristics are reported as median [interquartile] ([IQR]) and frequency (percentage) for continuous and categorical variables, respectively. Qualitative variables were compared using Chi-squared or Fisher's exact tests (as appropriate), while Mann–Whitney’s test was used for continuous variables. Patient subsets were differentiated based on the presence or not of other (besides VT) eosinophil-related organ involvements during the entire follow-up. After exclusion of patients with single-flare eosinophilia (*i.e.* parasitic or drug-induced eosinophilia) and those with less than two weeks of follow-up, predictors of relapse were identified using a Cox proportional hazards model. The final multivariate model was performed using a backward stepwise procedure including all variables with a p-value < 0.10 in univariate analysis. Results are expressed as hazard ratios (HR) and 95% confidence intervals (CI95%). Relapse-free status was analyzed using the Kaplan–Meier method, and compared using log rank tests. Tests are bilateral and p-values less than 0.05 were considered significant. All analyses were performed using SAS software (version 9.4; SAS Institute, Carry, NC, USA).

### Ethical and regulatory considerations

All methods were carried out in accordance with relevant guidelines and regulations (i.e. the Good Clinical Practice protocol, the Declaration of Helsinki principles and the MR004 French legislation regarding observational retrospective studies). This study was approved by Foch Hospital’s independent ethics committee (IRB00012437) and informed consent was obtained from all subjects or from all legal guardian or parents if patients under 18 years old.

## Results

### Patient identification and baseline characteristics

**Figure 1:**
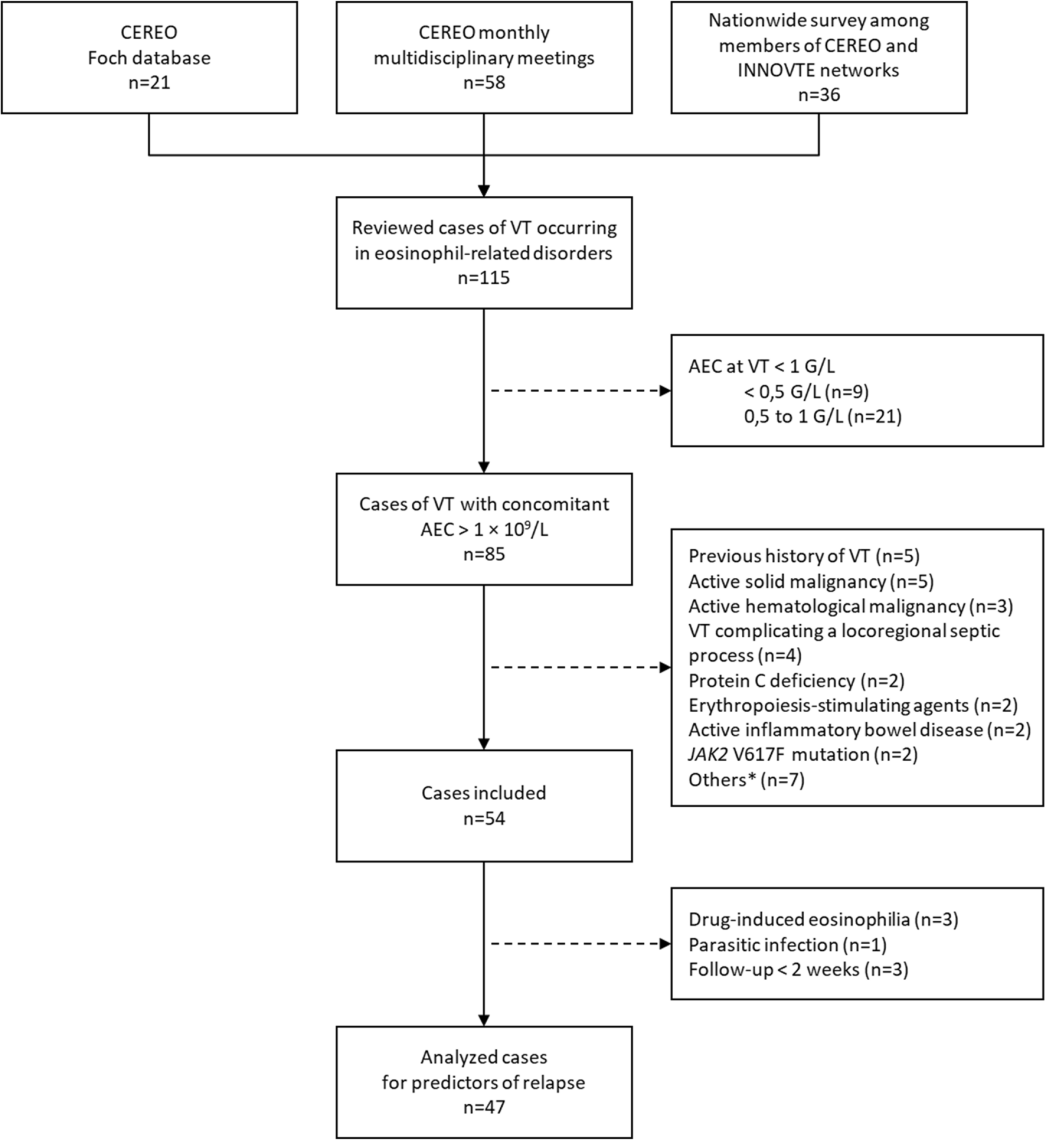
Flow-chart showing the search strategy and inclusion/exclusion criteria for the study population. AEC: absolute eosinophil count; CEREO: French national reference center for hyper-eosinophilic syndromes; INNOVTE: investigation network on venous thromboembolism; VT: venous thrombosis. *post-operative period, estrogen-based oral contraceptive therapy started within the previous six months, post-partum period; antiphospholipid syndrome, nephrotic syndrome, retinal vein occlusion, superficial venous thrombosis (a single patient each).

**Figure 2:**
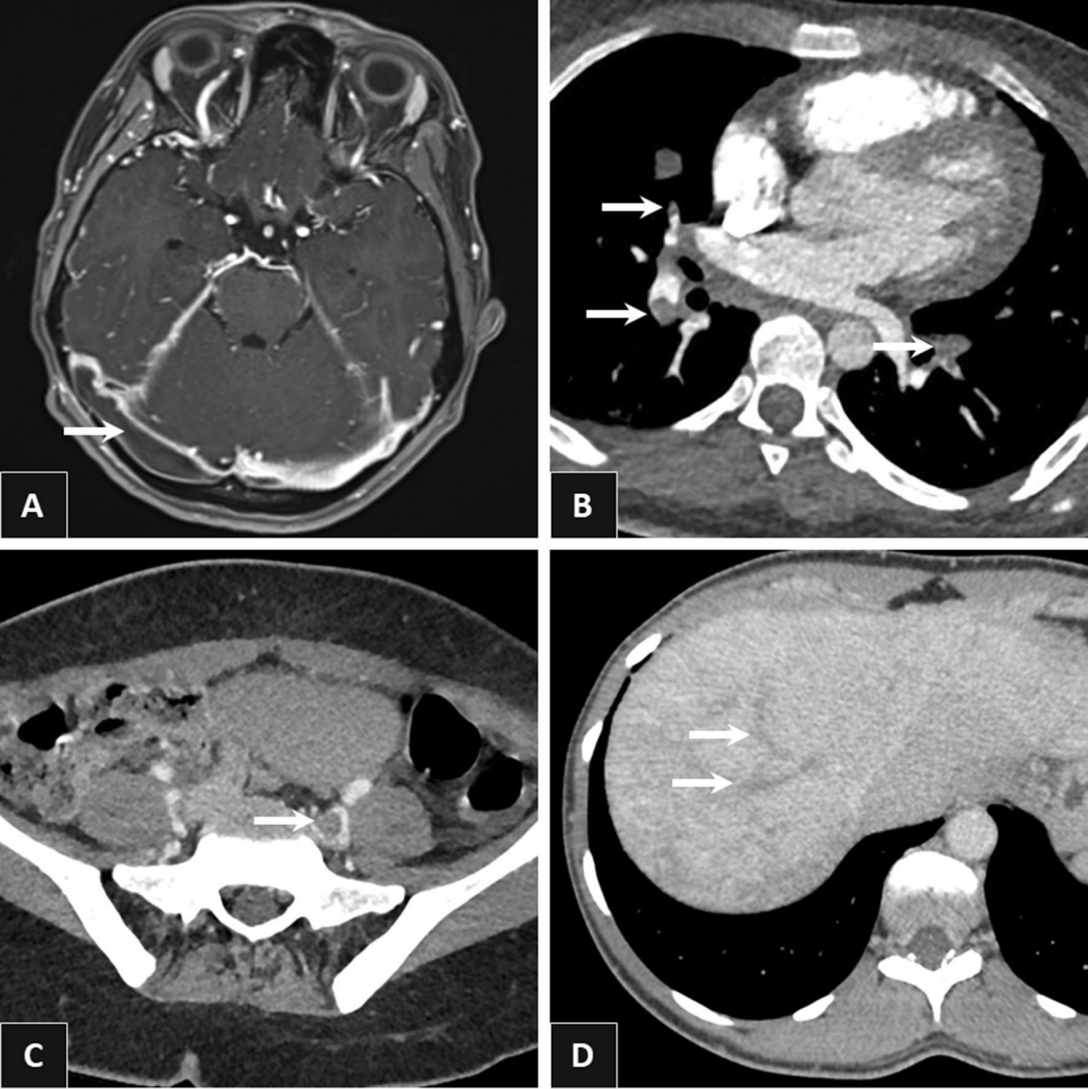
Various examples of eosinophil-related venous thrombosis. Right transverse sinus thrombosis on brain Magnetic Resonance Imaging (**A**), bilateral pulmonary embolism (middle lobe medial segment, right posterior basal segment left anterior basal segment) on Computed Tomography pulmonary angiography (**B**), left common iliac vein thrombosis on Computed Tomography venography (**C**), right and middle hepatic thromboses on contrast-enhanced abdominal Computed Tomography at late portal phase (**D**).

Among the 115 patients screened for eosinophilia and concomitant VT, 54 (47%) fulfilled the inclusion criteria (Fig. 1). Thirty-three (61%) were males and their median [IQR] age at VT onset was 54 [32–70] years. Eleven (20%) and 18 (33%) patients had either moderate (e.g. lung or urinary tract infections) or weak (e.g. arterial hypertension or diabetes mellitus) risk factors for VTE, respectively (Table 1). VT occurred between October 2001 and May 2020, despite ongoing treatment with anticoagulant (n = 1) and antiplatelet therapy (n = 4) in some patients, and was the initial manifestation of eosinophil-related organ involvement in 29 (54%) patients (including 14 (26%) patients with VT-restricted eosinophil-related organ involvement). Thirty-two (59%) patients had a single VT, while 22 (41%) patients had concomitant multiple VT at different anatomical sites. VT consisted mostly of pulmonary embolism (PE, n = 28; 52%, including seven who required admission to the intensive care unit), lower limb deep venous thrombosis (n = 20; 37%), hepatic (n = 6; 11%) and portal vein thromboses (n = 5; 9%) (Fig. 2). Two cerebral sinus thromboses occurred in patients with VT-restricted eosinophilic disorder.

**Table 1:** Demographic, clinical, and biological features of patients with venous thrombosis and eosinophilia.

	All patients n = 54	VT-restricted eosinophil-related organ involvement n = 14	Systemic eosinophil-related organ involvement n = 40	P-value
**Demographic data**				
Male	33 (61)	10 (71.4)	23 (57.5)	0.358
Age at VT (years)	54 [32 – 70]	62 [29 – 73]	51 [33 – 68]	0.567
**Comorbidities**				
BMI > 30 kg/m^2^	8 (15)	2 (14)	6 (15)	0.948
Active smoking	11 (20)	4 (29)	7 (17.5)	0.376
History of atopy	10 (18.5)	3 (21)	7 (17.5)	0.745
Arterial hypertension	12 (22)	3 (21)	9 (22.5)	0.943
Congestive heart failure	7 (13)	2 (14)	5 (12.5)	0.864
Diabetes mellitus	6 (11)	3 (21.4)	3 (7.5)	0.154
Autoimmune disease	12 (22)	3 (21)	9 (22.5)	0.934
Other risk factors for VTE				
≥ 1 moderate risk factor for VTE^a^	11 (20)	3 (21)	8 (30)	0.909
≥ 1 weak risk factor for VTE^a^	18 (33)	7 (50)	11 (27.5)	0.188
VT characteristics				
VT as first clinical manifestation	29 (54)	14 (100)	15 (37,5)	** < 0.001**
Multiple VT	22 (41)	8 (57)	14 (35)	0.147
Pulmonary embolism	28 (52)	7 (50)	21 (52.5)	0.872
Lower-limb DVT	20 (37)	8 (57)	12 (30)	0.070
Lower-limb DVT subtype				0.852
Proximal DVT	12 (22)	5 (36)	7 (17.5)	–
Distal DVT	8 (15)	3 (21)	5 (12.5)	–
Subhepatic vein thrombosis	6 (11)	1 (7)	5 (12.5)	0.583
Portal vein thrombosis	5 (9)	0 (0)	5 (12.5)	0.165
Cerebral venous sinus thrombosis	2 (4)	2 (14)	0 (0)	0.015
Others*	15 (28)	3 (21)	12 (30)	0.538
**Classification of eosinophil-related disorders**			0.161	
Clonal	6 (11)	1 (7)	5 (12.5)	–
Reactive	8 (15)	2 (14)	6 (15)	–
Lymphocyte variant HES	1 (2)	0 (0)	1 (2.5)	–
Overlapping**	9 (17)	0 (0)	9 (23)	–
Idiopathic	31 (57)	11 (79)	20 (50)	–
**Eosinophil-related organ involvements**				
Number of affected organs (besides VT)	1 [0.25–2]	–	2 [1,2]	NA
Lungs	17 (31.5)	–	17 (42.5)	NA
Eosinophilic asthma	11 (20)	–	11 (27.5)	NA
Eosinophilic pneumonia	9 (54)	–	9 (22.5)	NA
Eosinophilic pleural effusion	1 (2)	–	1 (2.5)	NA
Skin	14 (26)	–	14 (35)	NA
Thromboangiitis obliterans-like disease	3 (5.5)	–	3 (7.5)	NA
Biopsy-proven eosinophilic vasculitis	3 (5.5)	–	3 (7.5)	NA
Eosinophilic fasciitis	2 (4)	–	2 (5)	NA
Erythroderma	2 (4)	–	2 (5)	NA
HiIves	2 (4)	–	2 (5)	NA
Eosinophilic cellulitis (Well’s diseasesyndrome)	1 (2)	–	1 (2.5)	NA
Episodic angioedema with eosinophilia	1 (2)	–	1 (2.5)	NA
Kimura’s disease	1 (2)	–	1 (2.5)	NA
Eczema-like lesions	1 (2)	–	1 (2.5)	NA
Gastrointestinal tract	7 (13)	–	7 (17.5)	NA
Eosinophilic gastroenteritis	5 (9)	–	5 (12.5)	NA
Eosinophilic oesophagitis	2 (4)	–	2 (5)	NA
Eosinophilic cholangitis	1 (2)	–	1 (2.5)	NA
Lymph nodes	7 (13)	–	7 (17.5)	NA
Heart	7 (13)	–	7 (17.5)	NA
Eosinophilic myo(peri)carditis	6 (11)	–	6 (15)	NA
Endomyocardial fibrosis	1 (2)	–	1 (2.5)	NA
Intracardiac thrombus	2 (4)	–	2 (5)	NA
Arterial thrombosis	6 (11)	–	6 (15)	NA
Peripheral nervous system	5 (10)	–	5 (12.5)	NA
Mononeuritis	4 (7)	–	4 (10)	NA
Polyneuritis	1 (2)	–	1 (2.5)	NA
Central nervous system (stroke)	4 (7)	–	4 (10)	NA
Joints	2 (4)	–	2 (5)	NA
Kidney	1 (2)	–	1 (2.5)	NA
Urinary tract	1 (2)	–	1 (2.5)	NA
**Main biological features**				
AEC (G/L) at first VT	3.3 [1.6 – 7.4]	3.5 [1.4 – 10.4]	3.3 [1.6 – 7.4]	0.978
Peak AEC (G/L)	7 [3–14]	5.3 [3.2 – 20]	7.5 [3–14]	0.784
Polycythemia*** at first VT	1 (2)	1 (7)	0 (0)	0.088
Thrombocytosis**** at first VT	5 (9)	1 (7)	4 (10)	0.751
Neutrophilia***** at first VT	14 (26)	3 (21)	11 (27.5)	0.656
C-reactive protein at first VT (mg/L)	44.5 [8 – 66]	52 [11 – 68]	76 [38 – 76]	0.920
High total IgE levels	20/31 (64.5)	5/9 (56)	15/22 (68)	0.505
High tryptase levels	2/32 (6)	1/10 (10)	1/22 (5)	0.551
High vitamin B12 levels	7/25 (28)	2/6 (33)	5/19 (26)	0.739
*FIP1L1-PDGFRA* fusion gene	4 (7)	1 (7)	3 (7.5)	0.965
Aberrant T-cell population	3 (6)	0 (0)	3 (7.5)	0.560
**Initial treatment of VT**				
Anticoagulant therapy	52 (96)	13 (93)	39 (97.5)	0.429
Vitamin K antagonists	29/40 (72.5)	9/12 (75)	20/28 (71)	0.817
Direct oral anticoagulants	8/40 (20)	1/12 (8)	7/28 (25)	0.227
Low-molecular-weight heparin	3/40 (7.5)	2/12 (17)	1/28 (4)	0.150

Data are presented as no. (%) or median [IQR], unless otherwise specified.

AEC: absolute eosinophil count; BMI: body mass index; CKD-EPI: chronic kidney disease epidemiology collaboration; DVT: deep venous thrombosis; IQR: interquartile range; NA: not applicable; VT: venous thrombosis; VTE: venous thromboembolism.

*IgG4-related disease (n = 2), eosinophilic granulomatosis with polyangiitis (n = 6) and bullous pemphigoid (n = 1).

**mesenteric venous thrombosis, renal vein thrombosis, common iliac vein thrombosis, DVT of upper extremity, inferior vena cava thrombosis.

*** hemoglobin > 16.5 g/dL for males and > 16 g/dL for females.

**** platelet count > 400 G/L.

***** neutrophil count > 7.5 G/L.

^a^According to Konstantinides et al.^8^.

At VT onset, the median [IQR] AEC was 3.3 G/L [1.6 – 7.4]. Overall, 44/54 (81%) and 50/54 (93%) of the patients had eosinophilia ≥ 1.5 G/L (*i.e.* the common threshold used to define hypereosinophilia and subsequently HES in case of eosinophil-related organ involvement)^1^ either at the time of thrombosis or at least once during follow-up, respectively. Among the 40 (74%) patients with systemic eosinophil-related organ involvement (including five with concomitant treatment with corticosteroids at VT onset), the median number of affected organs besides VT was 2 [1, 2] and consisted mostly of lung (42.5%, e.g. eosinophilic asthma, n = 11; eosinophilic pneumonia, n = 9) and skin (35%, e.g. biopsy-proven eosinophilic vasculitis, n = 3; eosinophilic fasciitis, n = 2; eosinophilic cellulitis, episodic angioedema with eosinophilia, Kimura’s disease, a single patient each) involvements. At VT onset, 6 (11%) patients showed evidence of active arterial thrombosis (including stroke, upper and lower-limb distal ischemia, n = 2 each, with no evidence of cardiac involvement). Overall, the pathophysiological processes underlying eosinophilia were considered to be clonal (n = 6, 11%; including four patients with FIP1L1-PDGFRA-myeloid neoplasm with eosinophilia and two with chronic eosinophilic leukaemia not otherwise specified), reactive (n = 7, 13%; including three patients with drug-induced eosinophilia, two with cutaneous low-grade peripheral T-cell lymphoma, one with CD3 + 4–8-TCRab lymphocyte variant HES and one with parasitic infection), overlapping (n = 9, 17%, consisting of six patients with anti-myeloperoxydase (MPO) antineutrophil cytoplasm antibodies (ANCA)-negative EGPA, two with IgG4-related disease and one with bullous pemphigoid) and idiopathic (n = 31, 57%, including n = 29 patients fulfilling criteria for idiopathic HES^1^). Of note, none of the six patients with ANCA-negative EGPA showed signs of active vasculitic disease (e.g. purpura, mononeuritis multiplex or pauci-immune crescentic glomerulonephritis) at VT onset.

### Treatment regimens

Anticoagulant therapy (prescribed in all patients but one who presented with porto-sinusoidal vascular disease and received anticoagulants at a later stage of disease evolution) consisted of vitamin K antagonists, non-vitamin K antagonist oral anticoagulants (NOACs) or low-molecular-weight heparin in 29/40 (72.5%), 8/40 (20%) and 3/40 (7.5%) patients, respectively (missing data for 12 patients). No patient underwent systemic thrombolysis and two had hepatic vein angioplasty with stenting for Budd-Chiari syndrome. In the long run, other treatments included systemic corticosteroids (n = 48, 89%; including 9 patients who received high-dose (*i.e.* 120–1000 mg) initial pulses of methylprednisolone for 3–5 days), hydroxycarbamide (n = 10), imatinib mesylate (n = 7), cyclophosphamide (n = 7), methotrexate, azathioprine (n = 6 each), interferon alfa-2a, mepolizumab (n = 5 each), rituximab (n = 2), mycophenolate mofetil, infliximab and bexarotene (a single patient each).

### Outcomes

**Figure 3:**
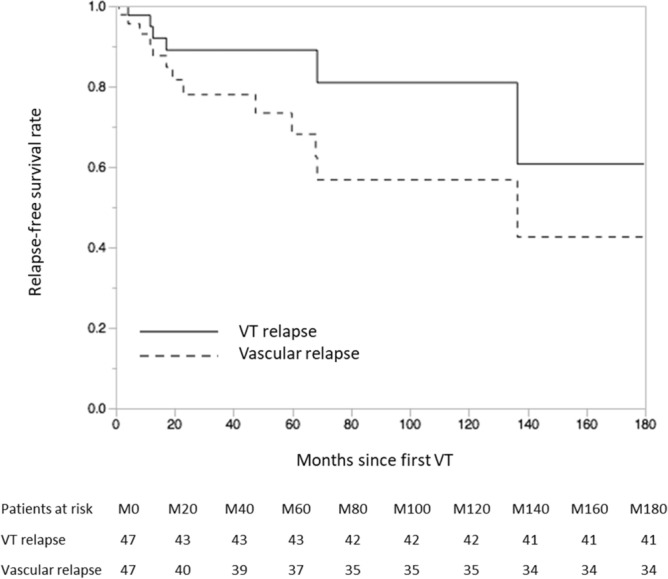
Kaplan Meier estimates of the relapse-free survival rates of both venous (solid line) and vascular (both venous and arterial) manifestations (dashed line). VT: venous thrombosis. Figure performed using SAS software (version 9.4; SAS Institute, Carry, NC, USA; https://www.sas.com/en_us/home.html).

After a median [IQR] follow-up of 24 [10 – 62] months since first VT, 7 (13%) patients (including two with concomitant arterial thrombosis) had a recurrence of VT, either at the same (n = 3) or at a different (n = 4) anatomical site than the initial episode, including 6 (86%) patients with persistent eosinophilia > 0.5 G/L (and 5 patients with AEC > 1G/L, while the remaining patient was an active smoker with arterial hypertension and BMI > 30 kg/m^2^) (Fig. 3). None of the latter patients had long-term anticoagulant therapy. None of the four patients with single-flare eosinophilia (drug-induced eosinophilia, n = 3; parasitic infection, n = 1) had recurrence of VT. Likewise, all 9 (17%) patients who presented with arterial thrombosis (including stroke, myocardial infarction, limb ischemia, n = 2 each; digital ischemia, retinal artery occlusion, non-arteritic anterior ischemic optic neuropathy, a single patient each) during follow-up had persistent eosinophilia > 0.5 G/L (including seven patients with AEC > 1G/L). Among the latter patients, three (33%) patients developed arterial thrombosis during follow-up despite long-term treatment with vitamin K antagonists (and International Normalized Ratio within the targeted range). No major bleeding was reported. Conversely, 15 of the 16 patients who discontinued anticoagulants while on sustained remission of their underlying eosinophil-related disease did not undergo VT relapse. Overall, two patients died during follow-up (bacterial pneumonia in a patient treated with corticosteroids and imatinib, and respiratory failure due to severe bronchospasm and PE in the setting of uncontrolled eosinophilia, a single patient each).

### Predictors of relapse

In univariate analysis, persistent eosinophilia was associated with a shorter time to VT relapse (HR 6.68; CI95% [3.25–16.57]; p = 0.002) while age, sex, other morbidities and risk factors for VTE, VT sites, eosinophil-related organ involvements, main biological features and concomitant treatments did not (data not shown). Conversely, long-term anticoagulant therapy showed a protective effect regarding the risk of subsequent VT relapse (HR 0.19; CI95% [0.01–0.74]; p = 0.027). In multivariate analysis, persistent eosinophilia was the sole variable associated with a shorter time to VT relapse (HR 7.48; CI95% [1.94–29.47]; p = 0.015). Likewise, persistent eosinophilia correlated with a shorter time to vascular relapse in both univariate (HR 16.58; CI95% [8.16–26.21]; p < 0.001) and multivariate analyses (HR 10.61; CI95% [1.58–17.96]; p = 0.019). Last, when considering only patients with eosinophilia ≥ 1.5 G/L at the time of venous thrombosis (n = 44), persistent eosinophilia was in multivariate analyses again the sole variable associated with both shorter times to venous (HR 7.33; CI95% [2.21–22.54]; p = 0.007) and vascular (HR 14.37; CI95% [1.32–8.91]; p = 0.041) relapses.

## Discussion

Despite growing interest in the basic molecular mechanisms underpinning eosinophil-related vascular toxicity, reported cases of arterial and venous thrombosis occurring in eosinophil-related diseases are scarce^12^. Our group previously reported on the first three cases of superficial thrombophlebitis revealing HES^13^. Here, after a stringent exclusion process of cases with moderate-to-strong risk factors for VT (besides eosinophilia), we report on various subtypes of VT (including highly unusual anatomical sites) occurring within the full-spectrum of eosinophil-related diseases (including clonal, reactive, overlapping and idiopathic eosinophilia) either as first disease manifestation or during follow-up. Moreover, we provide evidence suggesting that, in some patients, eosinophilia (whatever the underlying disease) could be a contributing factor to VT, and possibly warrant therapeutic intervention.

There is compelling evidence supporting the procoagulant effects of eosinophils. In mouse models, injury-induced venous thrombosis is dramatically reduced in either eosinophil-deficient or eosinophil-depleted mice^14^. At a basic level, eosinophils are potent producers of tissue factor^15^ and are able to generate procoagulant phospholipids and activate factor XII, all of which, both of which promote thrombin genesis via the intrinsic pathway^14^. Moreover, eosinophils are recruited in human thrombi^16^ and in atherosclerotic plaques where they are activated by platelets and in turn foster thrombus formation via the release of ECP, major basic protein (MBP), eosinophil peroxidase^17^ and platelet activation factor^18^. Likewise, recently discovered MBP-enriched eosinophil extracellular DNA traps also contribute to platelet activation^19,20^. Lastly, MBP’s ability to bind to thrombomodulin (and thereby to impair its anticoagulant effects)^21,22^, increased vascular permeability, as well as direct tissue and endothelial damage prompted by the shedding of cytotoxic granules as well as pro-inflammatory mediators are other potential factors contributing to an eosinophil-induced procoagulant state^12^. In the present series, since all six patients with EGPA (including a patient with ongoing treatment with cyclophosphamide) tested negative for MPO-ANCA and that none had signs of active vasculitic manifestations (either at VT onset or at vascular relapse) suggests that VT could be the consequence of eosinophil toxicity rather than active vasculitis.

In their 2007 review of HES-related cardiovascular manifestations, Ogbogu et al. mainly focused on cases of intracardiac thrombus and subsequent peripheral arterial emboli^3^. Overall, VT is poorly reported in the main series of patients with HES (whether clonal^23^, lymphocytic^24^ or idiopathic^7,25^) and EGPA is the sole eosinophil-related disease for which estimated rates of the prevalence of VT have been reported (ranging from 5 to 30% within series, and possibly higher in ANCA-negative patients)^26,27^. More recently, Maino et al. reviewed published cases of thrombotic (including venous, arterial and mixed) events occurring in HES (n = 124), EGPA (n = 80) or parasitic infestations (n = 22), yet neither AEC at thrombosis nor long-term outcomes were reported^28^. Here, we report on a wide variety of venous thrombotic manifestations (including patients with VT-restricted clinical presentation) involving multiple anatomical sites and with no clear correlation between the clinical picture of VT and underlying eosinophil-related diseases. Of note, some patients had active life-threatening disease (leading to one death), including cerebral venous sinus thrombosis, massive pulmonary embolism and catastrophic antiphospholipid syndrome-like presentation (in a patient who was initially treated with plasma exchanges due to yet unrecognized diagnosis of HES). Hence, as with any context of eosinophil-related organ damage, patients presenting with VT and eosinophilia should undergo a step-by-step individualized etiological workup seeking for the underlying condition leading to eosinophilia In order to avoid significant diagnostic delay^2,29^.

**Figure 4:**
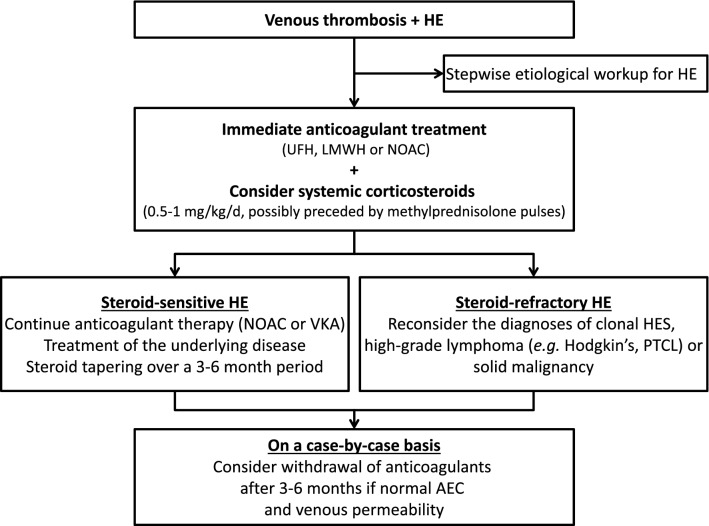
Suggested algorithm for the management of eosinophil-related venous thrombosis. AEC: absolute eosinophil count; HE: hypereosinophilia; LMWH: low-molecular-weight heparin; NOACs: non-vitamin K antagonist oral anticoagulants; PTCL: peripheral T-cell lymphoma; UFH: unfractionated heparin; VKAs: vitamin K antagonists.

No specific guidelines are available regarding the management of VT occurring in patients with eosinophilia. Contrary to antiphospholipid syndrome (another cause of dysimmune acquired thrombophilia)^30^, these preliminary data do not seem to address any worrisome signal regarding the use of NOACs in the setting of eosinophil-related VT, yet the low sample size precludes drawing any definite conclusions on this issue. Besides anticoagulant therapy, given the ability of corticosteroids to induce rapid normalization of AEC in most cases (with the notable exceptions of clonal eosinophilic disorders and eosinophilia related to high-grade lymphoma), initiation of systemic corticosteroids (e.g. 0.5–1 mg/kg of daily prednisone, possibly preceded by methylprednisolone pulses in case of life or organ-threatening VT) should be considered. In the long run, the optimal duration of anticoagulant therapy is unknown and the European Respiratory Society and European Federation of Internal Medicine-endorsed EGPA Consensus Task Force highlights that “*it is unknown whether anticoagulation should be prolonged in selected patients with persistent or recurring disease activity*“^31^. Overall, our data suggest that long-term normalization of AEC (owing to appropriate treatment of the underlying disease) could be of paramount importance, likely to prevent both VT and vascular relapses. Moreover, since 15 of the 16 patients who discontinued anticoagulants while on sustained remission of their underlying disease did not relapse, our data also suggest that anticoagulants could possibly be discontinued on a case-by-case basis in patients with long-term normalization of their AEC, taking into account other underlying risk factors and specificities inherent to the anatomical site involved (Fig. 4)^32,33^. Lastly, given the high rates of arterial thrombosis reported in patients with otherwise no overt cardiovascular risk factors, these data also suggest that general cardiovascular risk factors should be adequately managed.

This study has several drawbacks. First, up to 50% of initial reviewed cases were excluded from final analyses and some patients were cared for in tertiary referral centers for rare conditions (including HES, vasculitis and vascular liver diseases), both of which might have led to a selection bias. Yet, given the stringent exclusion criteria that were applied, we intentionally did not include a substantial number of additional cases with broad causes of eosinophilia (e.g.* STAT3*-mutated hyper-IgE syndrome or polyarteritis nodosa), thereby illustrating the diversity of situations where eosinophils are, at least partly, involved in venous thrombosis. Next, owing to the rarity of eosinophil-related diseases, the sample size and the number of events during follow-up were small and, despite careful selection of cases, up to half of the patients still had underlying low general risk factors for VTE (e.g. older age or active smoking). Last, given the retrospective design of the study, we were unable to assess whether, besides AEC, other biological parameters (including markers of eosinophil activation and degranulation) could also correlate with outcomes.

Regardless of these limitations, this study—the first longitudinal analysis dedicated to VT occurring in eosinophil-related diseases—further emphasizes the fact that eosinophilia (whatever the underlying disease) is a potent thrombogenic factor. It provides useful data for physicians involved in the field of eosinophil-related disorders and suggests that, in a subset of patients with otherwise unexplained VT and eosinophilia, long-term normalization of AEC could prevent the recurrence of VT. Further large-scale studies are needed in order to confirm these preliminary findings.

## Supplementary information

Supplementary information.
