# Supplementary material for: Venous thrombosis and predictors of relapse in eosinophil-related diseases
Source: Sci Rep. 2021 Mar 18;11:6388. doi: 10.1038/s41598-021-85852-9 (PMC7973521; doi:10.1038/s41598-021-85852-9)
Supplement: Supplementary file 1 — Supplementary information. [file 41598_2021_85852_MOESM1_ESM.docx]

**Venous thrombosis and predictors of relapse in eosinophil-related diseases**

Valériane Réau^1,2^, Alexandre Vallée^3^, Benjamin Terrier^4^, Aurélie Plessier^5^, Noémie Abisror^6^, Félix Ackermann^2-7^, Ruben Benainous^8^, Gérôme Bohelay^9^, Marie‑Laure Chabi-Charvillat^10^, Divi Cornec^11^, Anne‑Claire Desbois^12^, Stanislas Faguer^13^, Nathalie Freymond^14^, Antoine Gaillet^2,7^, Mohamed Hamidou^15^, Martin Killian^16^, Sylvain Le Jeune^8^, Anne Marchetti^17^, Guy Meyer^18^, Francisco Osorio-Perez^19^, Kewin Panel^2-7^, Pierre-Emmanuel Rautou^5^, Julien Rohmer^2,7^, Nicolas Simon^20^, Colas Tcherakian^21^, Marc Vasse^22^, Elina Zuelgaray^23^, Guillaume Lefevre^2,24^, Jean‑Emmanuel Kahn^2-25^, Matthieu Groh^2,7^*

**Affiliations**

^1^ Department of Internal and Geriatric medicine, Henri Mondor Hospital, Assistance Publique-Hôpitaux de Paris, Créteil, France.

^2^ National Referral Center for Hypereosinophilic Syndromes (CEREO), France.

^3^ Department of Clinical Research and Innovation (DRCI), Hôpital Foch, 92150 Suresnes, France.

^4^ Department of Internal medicine, National Referral Center for Systemic and Autoimmune Diseases, Cochin Hospital, Assistance Publique-Hôpitaux de Paris, Paris, France.

^5^ Department of Hepatology, Beaujon Hospital, Assistance Publique-Hôpitaux de Paris, Clichy, France.

^6^ Department of Internal medicine, Saint Antoine Hospital, Assistance Publique-Hôpitaux de Paris, Paris, France.

^7^ Department of Internal medicine, Foch Hospital, Suresnes, France.

^8^ Department of Internal medicine, Avicenne Hospital, Assistance Publique-Hôpitaux de Paris, Bobigny, France.

^9^ Department of Dermatology, Avicenne Hospital, Assistance Publique-Hôpitaux de Paris, Bobigny, France.

^10^ Department of Radiology, Foch Hospital, Suresnes, France.

^11^ Department of Rheumatology, Brest University Hospital, Brest, France.

^12^ Department of Internal medicine, Pitié-Salpêtrière Hospital, Assistance Publique-Hôpitaux de Paris, Paris, France.

^13^ Department of Nephrology, Toulouse University Hospital, Toulouse, France.

^14^ Department of Pulmonology, Lyon University Hospital, Lyon, France.

^15^ Department of Internal medicine, Hôtel-Dieu University Hospital, Nantes, France.

^16^ Department of Internal medicine, Saint-Etienne University Hospital, Saint-Etienne, France.

^17^ Department of Dermatology, Lyon-Sud Hospital, Pierre-Bénite, France.

^18^ Pulmonology and Intensive Care Service, Georges Pompidou European Hospital, Assistance Publique-Hôpitaux de Paris, Paris, France.

^19^ Department of Internal medicine, Dax-Côte d'Argent Hospital, France.

^20^ Department of Internal medicine, Grenoble Alpes University Hospital, Grenoble, France.

^21^ Department of Pulmonology, Foch Hospital, Suresnes, France.

^22^ Department of Clinical Biology, Foch Hospital, Suresnes, France; UMR-S INSERM 1176 Université Paris-Saclay, Le Kremlin-Bicêtre, France.

^23^ Department of Dermatology, Saint Louis, Hospital, Assistance Publique-Hôpitaux de Paris, Paris, France.

^24^ Department of Internal medicine, Lille University Hospital, Lille, France.

^25^ Department of Internal medicine, Ambroise Paré Hospital, Assistance Publique-Hôpitaux de Paris, Boulogne-Billancourt, France.

**Corresponding author**

Matthieu Groh, Department of Internal medicine, National Reference Center for Hypereosinophilic Syndromes (CEREO), Hôpital Foch, 40, rue Worth, 92151 Suresnes Cedex, France; m.groh@hopital-foch.com; Phone: +33 146253100; Fax: +33 146252783

**Supplementary Appendix: comprehensive list of study exclusion criteria**

Exclusion criteria were either:

1. Prior history of VT;
2. Hereditary thrombophilia, *e.g.* either factor V Leiden or prothrombin G20210A homozygous (or double heterozygous) mutations, deficiency of antithrombin (activity level < 80%), protein C (activity level < 60%) or protein S (activity level < 40%), hypofibrinogenemia (< 1 g/L) and hyperhomocysteinemia (> 30 µmol/L);
3. Conditions or comorbidities leading to acquired thrombophilia, including pregnancy or the post-partum period (up to 6 weeks after delivery), post‑operative period (less than one month), antiphospholipid syndrome (as defined by the Sidney criteria^1^), Behçet’s disease (as defined by the International Criteria for Behçet's Disease^2^), active inflammatory bowel disease, nephrotic syndrome with hypoalbuminemia (< 20 g/L), active solid or hematological malignancy (with the exclusion of non-melanoma skin cancers, low-grade peripheral T-cell lymphomas), spinal cord injury, thrombocytosis (> 1.000 G/L), polycythemia (> 16.5 g/dL in men, > 16 g/dL in women), *JAK2*V617F mutation, Coronavirus disease 2019;
4. Concomitant treatment with either estrogen-based oral contraceptive therapy (started within the last six months prior to the first venous thrombosis event), hormone replacement therapy (started within the last two years prior to the first venous thrombosis event), any hormone therapy during medically-assisted procreation, erythropoiesis-stimulating agents, immunomodulatory imide drugs;
5. Other major (relative risk (RR) > 10) transient or reversible predisposing factor for VTE according to the European Society of Cardiology and European Respiratory Society^3^ *i.e.* history of either lower limb fracture, hospitalization for heart failure or atrial fibrillation/flutter, hip or knee replacement, major trauma or myocardial infarction, within the last three months.

References

1. Miyakis S, Lockshin MD, Atsumi T, et al. International consensus statement on an update of the classification criteria for definite antiphospholipid syndrome (APS). J Thromb Haemost 2006;4:295‑306.

2. International Team for the Revision of the International Criteria for Behçet’s Disease (ITR-ICBD). The International Criteria for Behçet’s Disease (ICBD): a collaborative study of 27 countries on the sensitivity and specificity of the new criteria. J Eur Acad Dermatol Venereol 2014;28:338‑47.

3. Konstantinides SV, Meyer G, Becattini C, et al. 2019 ESC Guidelines for the diagnosis and management of acute pulmonary embolism developed in collaboration with the European Respiratory Society (ERS). Eur Heart J 2020;41:543‑603.
